# Graphitic Nanocup Architectures for Advanced Nanotechnology Applications

**DOI:** 10.3390/nano10091862

**Published:** 2020-09-17

**Authors:** Hyehee Kim, Sen Gao, Myung Gwan Hahm, Chi Won Ahn, Hyun Young Jung, Yung Joon Jung

**Affiliations:** 1 Department of Mechanical and Industrial Engineering, Northeastern University, Boston, MA 02115, USA; kim.hye@northeastern.edu (H.K.); gao.se@northeastern.edu (S.G.); 2 Department of Materials Science and Engineering, Inha University, 100 Inharo, Michuhol-gu, Incheon 22212, Korea; mghahm@inha.ac.kr; 3 National Nanofab Center, KAIST, 291 Daehak-Ro, Yusung-Gu, Daejeon 34141, Korea; cwahn@nnfc.re.kr; 4 Department of Energy Engineering, Gyeongnam National University of Science and Technology, Jinju-si, Gyeongnam 52725, Korea; hyjung@gntech.ac.kr

**Keywords:** graphitic nanoscale architecture, precisely controllable nanostructure, flexible and transparent supercapacitor, carbon nanocup container

## Abstract

The synthesis of controllable hollow graphitic architectures can engender revolutionary changes in nanotechnology. Here, we present the synthesis, processing, and possible applications of low aspect ratio hollow graphitic nanoscale architectures that can be precisely engineered into morphologies of (1) continuous carbon nanocups, (2) branched carbon nanocups, and (3) carbon nanotubes–carbon nanocups hybrid films. These complex graphitic nanocup-architectures could be fabricated by using a highly designed short anodized alumina oxide nanochannels, followed by a thermal chemical vapor deposition of carbon. The highly porous film of nanocups is mechanically flexible, highly conductive, and optically transparent, making the film attractive for various applications such as multifunctional and high-performance electrodes for energy storage devices, nanoscale containers for nanogram quantities of materials, and nanometrology.

## 1. Introduction

**Figure 1:**
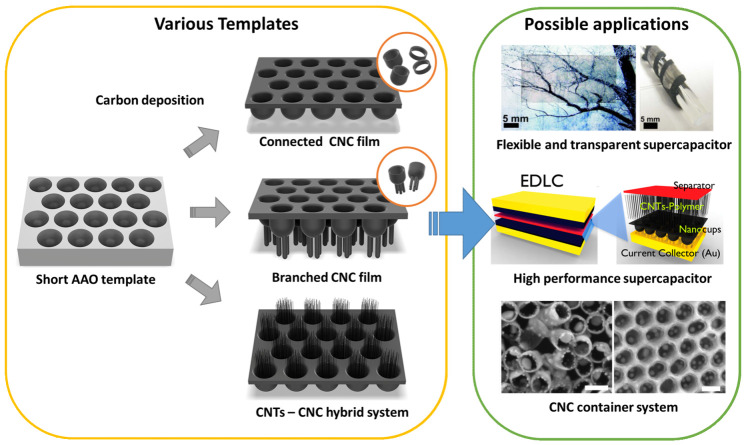
Schematic of various architectures using the anodized aluminum oxide (AAO) template and their applications: flexible and transparent supercapacitors [37], high-performance supercapacitors [36], and carbon nanocups (CNC) container system [27]. Insert images were reproduced with permission from [27,36,37]. Copyright (2009) American Chemical Society, (2012) American Chemical Society, and (2012) Springer Nature.

For decades, diverse graphitic nanostructures (e.g., nanographite, carbon nanotubes, carbyne, and graphene) have been actively researched for applications in energy storage devices, bio/chemical sensors, drug delivery, etc. [1,2,3,4,5,6,7,8,9,10,11,12,13,14]. While significant progress has been made on both developing large-scale synthesis and unveiling their exceptional properties, there remain challenges in controlling morphology and proportion of individual nanoscale units, which limits their use in down-to-earth applications [15,16,17,18,19,20,21,22]. Particularly one-dimensional (1-D) graphitic structures (e.g., carbon nanotubes) are attractive in that other nanomaterials or functional molecules can be trapped or embedded inside them for advanced multicomponent systems [23,24,25]. However, it is difficult to effectively intercalate abundant nanomaterials such as nanoparticles, drugs, or polymers within the hollow tubular structure where length/diameter (L/D) aspect ratios often range between 10^3^ and 10^5^ [25,26]. Fortunately, the recent flurry of activity successfully introduced a unique cup-shaped carbon nanostructure that allows unprecedented control over structural morphology and dimension and easy insertion of nanomaterials inside them [27]. This review covers the synthesis and processing of hollow carbon nanostructures and demonstrations of their potential applications. This hollow carbon nanostructured film was fabricated by precisely tailoring the nanopores of the anodized aluminum oxide (AAO) template, which has been widely used to form a dense array of nanochannels [28,29,30,31,32,33,34,35]. The AAO template allows precise control of nanoscale pores such as the length, width, and diameter [21,27,36,37,38,39,40,41,42], enabling the design of cup-shaped nanostructures with low aspect ratio. By depositing carbon materials on this AAO template, low aspect ratio graphitic nanoscale cup structures can be fabricated, resulting in a film of uniformly arranged carbon nanocups (see Figure 1). The deposition of the graphitic carbon film is performed by a high-temperature chemical vapor deposition (CVD) process, and the thickness of the nanocups film could be controlled by changing the CVD reaction time. The unique carbon nanostructured cups are continuously and uniformly connected like a thin film of honeycombs. The film of connected nanocups exhibits high specific surface area and enables fast electron transfer [43,44]. Furthermore, it is mechanically flexible and optically transparent. Shape control is not only limited to the dimensions but also in terms of fabricating branches on the bottom of the nanocups or growing vertically aligned CNTs inside the cups by employing a thermal CVD process. This connected nanocups film is an ideal template to build a multicomponent system, as other nanomaterials, molecules, or polymers can be easily inserted and evenly distributed over the cups [27,36,37,38,45,46].

Figure 1 shows a schematic of various carbon nanocups (CNC) architectures and their potential applications. CNC diameter and length can be controlled to optimize the function of the CNC film. We review three CNC-based nanostructures: (1) thin CNC film, (2) branched CNC film, and (3) CNTs–CNC hybrid structure. These structures are useful for applications that require optical transparency, mechanical flexibility, and structural continuity. In this paper, we cover (1) flexible and transparent supercapacitors, (2) high-performance supercapacitors, and (3) CNC container system that can accommodate nanoparticles, polymers, or liquid droplets.

## 2. Fabrication and Modification of Graphitic Nanoscale Architectures

### 2.1. Carbon Nanocups (CNC) Thin Film

**Figure 2:**
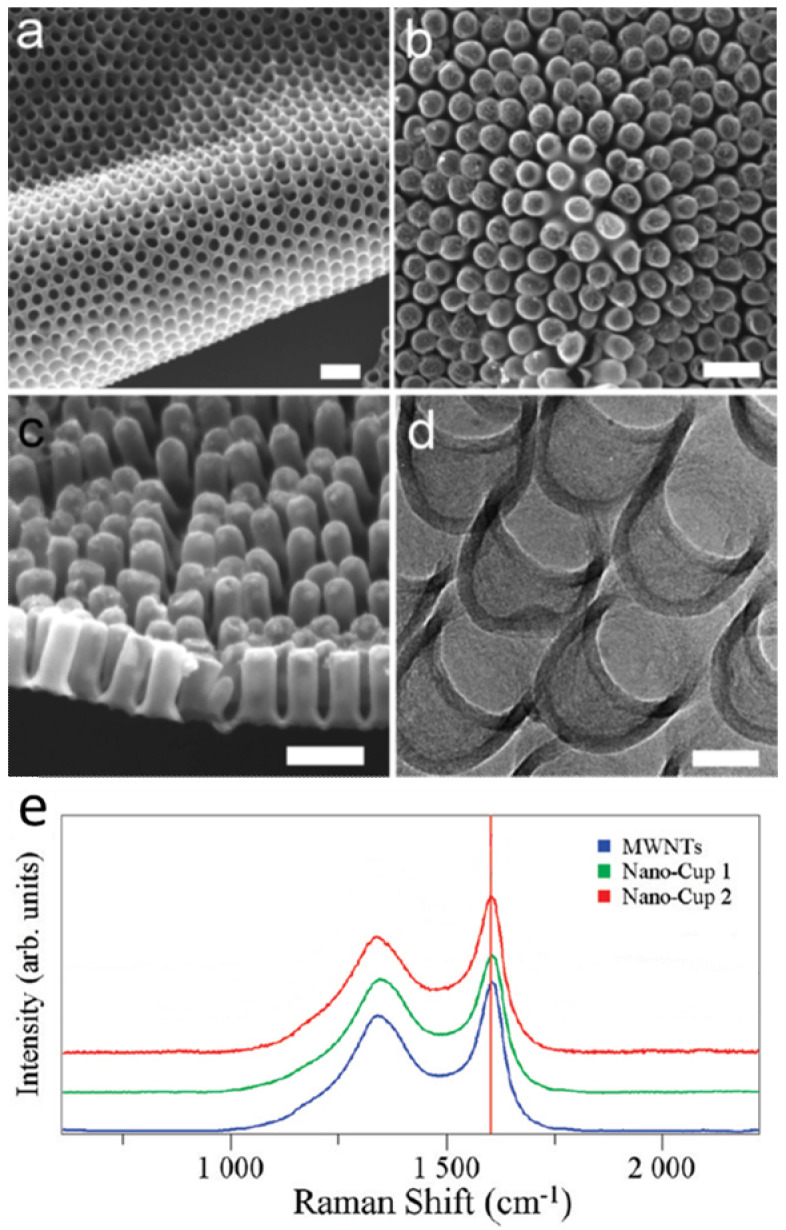
SEM and TEM micrographs of a two-dimensional carbon nanocup film structure after removing the AAO template. SEM images show (**a**) the upside of highly dense carbon nanocup arrays connected with a thin graphite layer, (**b**) a two-dimensional and flexible film of carbon nanocups, and (**c**) the side view of carbon nanocups (100 nm diameter and 200 nm length) connected with a graphitic layer of 10 nm thicknesses. Scale bars are 200 nm. (**d**) A TEM image shows connected arrays of carbon nanocup film with 80 nm diameter and 80 nm length. Scale bar is 50 nm. (**e**) Raman spectra taken from MWNTs (10 μm in length), long nanocups (180 μm in length), and short nanocups (60 nm in length). Reproduced with permission from [27]. Copyright (2009) American Chemical Society.

Carbon nanocups (CNC) thin film is an array of carbon nanocups with a short aspect ratio of 1–2 (Figure 2a,b). This two-dimensional film contains a well-organized array of nanocups where the uniform density results in high porosity. The structure of the nanocups can be precisely controlled in terms of their length, diameter, and wall thickness. Figure 2c shows the scanning electron microscopy (SEM) image of a CNC film with an L/D aspect ratio of 2, which has a 100 nm diameter and 200 nm length. Figure 2d shows the shorter CNCs with an aspect ratio of 1 with 80 nm diameter and 80 nm length. The thickness of nanocups shown in Figure 2 is 10 nm, and can be easily controlled by the time of carbon deposition or the concentration of carbon source. The CNC film demonstrates attractive features such as ease of structure control, high porosity, excellent flexibility, and high mechanical strength [27,37].

The CNC film is fabricated using the AAO template that was fabricated by a two-step anodization process. A high-purity Al foil (Alfa Aesar, 99.99%) was first anodized at 40–45 V for 4 h in 3–5% oxalic acid (C_2_H_4_O_2_) solution at room temperature. Then, the anodized film was dipped into an acid mixture solution (5% of phosphoric and 5% of chromic acid) for 24 h. After the removal of the first anodized aluminum layer, the second anodization, which plays a crucial role in controlling the dimension of CNC, was performed. To fabricate the shorter aspect ratio CNC, the second anodization was performed for extremely short time of 20–40 s, and this resulted in 80–200 nm of length. After that, the sample was soaked into the phosphoric acid again for 1 h to widen the diameter of the nanochannel. After the preparation of a well-controlled AAO template, the carbon was deposited on the template by a chemical vapor deposition (CVD) using acetylene as a carbon source. After the deposition of the graphitic carbon layer on the AAO template, the sample was soaked in 33% of the hydrofluoric acid solution to remove the AAO template. Finally, a self-standing and flexible thin CNC film was achieved.

Raman spectroscopy was performed using a 532 nm laser probe to investigate the lattice structure and graphitization of the nanocup structure. The disorder-induced D band is an indicator of presence of defects while the G band (~1600 cm^−1^) is associated with the tangential modes of the graphene structure. The D and G bands have been observed in the spectral range of 1200–1700 cm^−1^ [47,48]. Figure 2e shows the Raman spectra obtained from multiwalled carbon nanotubes (MWNTs) with 10 μm length and nanocups of similar diameter that have different lengths of 180 μm (nanocup 1) and 60 nm (nanocup 2). We have observed that the peak intensity ratios (I_D_/I_G_) are about 0.41 and 0.45 for long and short CNCs, respectively, comparable to that of long MWNTs (10 μm in length, I_D_/I_G_ = ~0.32). This result indicates that the degree of disorder of the carbon nanocup structures is comparable to that of MWNTs.

### 2.2. Branched Carbon Nanocups (B-CNC) Thin Film

**Figure 3:**
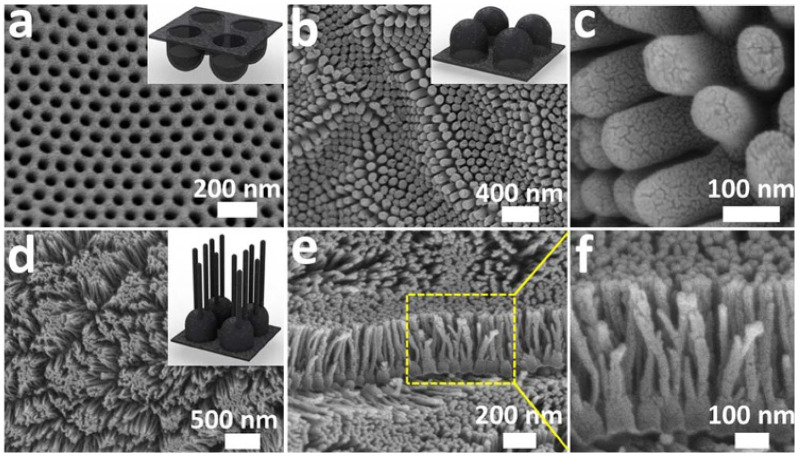
SEM images of CNC. SEM images of (**a**) concave and (**b**,**c**) convex, and (**d**–**f**) branched nanocup films. (**b**) SEM image of convex nanocup film with 80 ± 10 nm in diameter and 140 ± 10 nm in length, and (**c**) shows a high magnification of (**b**). (**e**,**f**) Cross-sectional views of (**d**) branched nanocup film, and (**f**) is a high magnification image of (**e**), where short carbon nanotubes (25 nm in diameter and 330 ± 10 nm in length) are branched from the bottom of a nanocup. The inset figures, respectively, show schematics of concave, convex, and branched convex nanocup film. Reproduced with permission from [37]. Copyright (2012) Springer Nature.

Figure 3 shows SEM images of CNCs (3a−c) and branched CNCs (3d−f). To fabricate branched CNCs, CNCs were first fabricated with channels of 80 ± 10 nm in diameter and 140 ± 10 nm in length. Afterwards, branches were developed at the bottom of the nanocups with 25 nm in diameter and 330 ± 10 nm in length. Such branched CNCs maximize the surface area of the thin film, and this highly dense and ordered array of nanocups with continuous graphitic thin film can be an ideal electrode material for energy storage devices. This branched CNC thin film shows attractive features such as excellent flexibility and strong mechanical strength, precisely controllable structure, and even improved porosity with a large surface area.

The fabrication of branches on the bottom of CNC follows the same procedure as CNC film fabrication (Section 2.1). After an hour of widening process, a third anodization is carried out for 5 min at 25 V in a 3% oxalic acid solution. The graphitic carbon deposition by CVD using acetylene was then followed, resulting in the production of a continuous film of branched nanocups. The surface area of the branched CNC film was calculated as 63 m^2^/g, while CNC film (convex type) showed surface area of 49 m^2^/g. When this branched CNC film was used as an electrode, the surface area exposed to the electrolyte was 2.3 times higher than the typical convex CNC film. Further, this film showed excellent electrical conductivity of 117 S/m, which is higher than the regular activated carbon electrode [49].

### 2.3. Carbon Nanotubes (CNTs)–CNC Hybrid Structure

**Figure 4:**
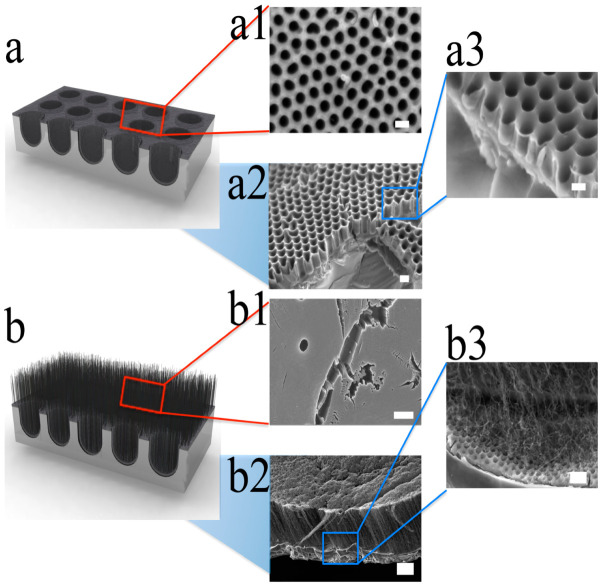
Schematic drawing of SEM images from CNCs and CNTs−CNC hybrid structure. (**a**) Schematic illustration of CNC. (**a1**) SEM image on the top surface of CNC, (**a2**) a low-magnification SEM of the cross-sectional view, and (**a3**) high-magnification cross-sectional images. The scale bars are 200, 200, and 120 nm, respectively. The images clearly show hollow structures with low aspect ratio. (**b**) Schematic of vertically aligned CNTs grown on the surface of CNC. (**b1**) A top-view SEM image of vertically aligned CNTs, (**b2**) a side view of low-magnification SEM image, and (**b3**) high-magnification SEM image of a vertically aligned CNTs−CNC structure. The scale bars are 200, 4, and 400 nm, respectively. Reproduced with permission from [36]. Copyright (2012) American Chemical Society.

The hybrid structure of CNTs and CNCs was developed to increase the surface area to be even greater than the branched CNC film. The vertically aligned CNTs were grown on the CNC by two steps of the CVD process. Figure 4a shows SEM images of the well-organized and interconnected CNCs with low aspect ratio, and Figure 4b shows CNTs–CNC hybrid structure. CNC film was prepared by AAO nanochannels and catalyst-free CVD process, as explained in Section 2.1. To fabricate CNTs–CNC hybrid structure, 1.5 nm of iron was deposited on the surface of the prepared CNC layer as a catalyst for CNTs growth. Then, vertically aligned CNTs were grown by a water-assisted CVD technique [50,51]. The water-assisted CVD process did not exceed 650 °C to avoid melting of AAO template. This second CVD process resulted in highly dense vertically aligned CNTs with 5–10 μm of length on the porous CNC structure (Figure 4b).

The specific surface area of CNC arrays was measured by surface adsorption analysis using the Brunauer–Emmett–Teller (BET) theory, and it was 810 m^2^/g. The high porous CNC film showed larger specific surface area than the experimentally synthesized CNTs (700–790 m^2^/g) [52,53]. The CNC film itself had high specific surface area, but the CNTs–CNC hybrid structure showed even higher specific surface area of 1340 m^2^/g. The increased surface area of the CNTs–CNC hybrid structure indicated that vertically oriented CNT walls provide surface exuberance towards the nanocups. The CNTs–CNC hybrid structure provides higher surface area than CNTs itself [36,52] and has a continuous CNC film on the bottom that increases electrical conductivity.

## 3. Branched CNC for Electrodes of Flexible and Transparent Supercapacitors

A branched CNC film can be used as a nanofabricated carbon electrode that has a porous template with complex shape and a porous interconnected array mold to create a mechanically flexible and optically transparent thin-film solid supercapacitor. The elegantly textured graphite film acts as an electrode, and a current collector acts as an integrated thin-film supercapacitor with the solid polymer electrolyte. The unique morphology of a nanostructured electrode and conformal electrolyte packages are powerful in providing sufficient energy and power density for energy storage devices, in addition to their excellent mechanical flexibility and optical transparency.

The branched CNC film has a two-dimensional structure with an extremely large surface area, which is an attractive feature for electrodes. The length of branches was optimized to maximize the interfacial area between electrodes and polymer electrolytes and also controlled to obtain optical transparency. The branched CNC film has 2.3 times greater interfacial area exposed to the electrolyte than that of normal CNC films. The branched CNC film is also unique in that it has well-organized nanocups in the innermost layer similar to normal CNC films. In contrast, the outmost layer, i.e., surface of branches, shows a defective and a forest-like surface (Figure 3). These defects of the branches can be reactive sites and serve as interfaces between electrode and electrolyte resulting in effective charge transfer.

**Figure 5:**
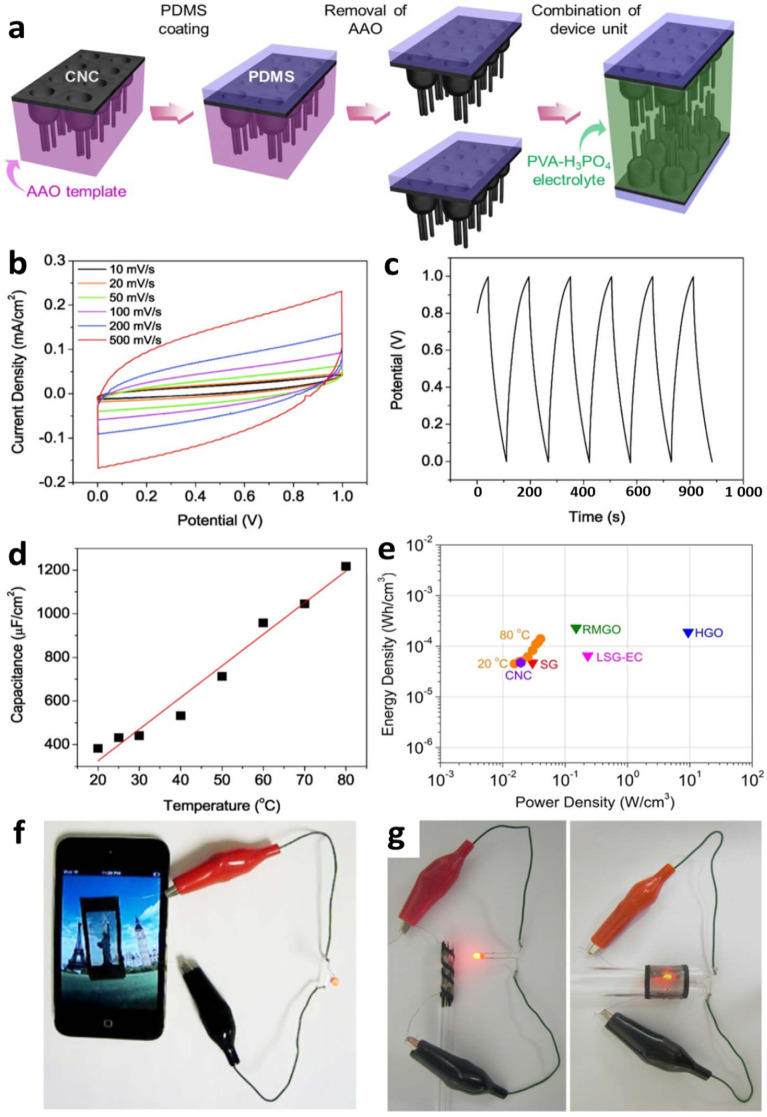
(**a**) Schematics of the fabrication process of a branched CNC-based supercapacitor. (**b**–**e**) Electrochemical properties of branched CNC supercapacitor devices: (**b**) cyclic voltammetry (CV) measured with 10–500 mVs^−1^ scan rates. (**c**) Galvanostatic charge/discharge (CD) results measured at constant current density of 5 μAcm^−2^. (**d**) The capacitance changes as a function of temperature (20 to 80 °C). (**e**) Ragone plot (SG: single-layer graphene [54], RMGO: reduced multilayer graphene oxide [54], HGO: hydrated graphitic oxide [56], LSG-EC: laser-scribed graphene electrochemical capacitor [55]). (**f**,**g**) Optical pictures demonstrating optical transparency and mechanical flexibility of a large-scale CNC supercapacitor films. Reproduced with permission from [37]. Copyright (2012) Springer Nature.

The transparent and flexible supercapacitors were fabricated by impregnating branched CNC film electrodes in transparent polymer electrolyte films (Figure 5a). The branched CNC film was transferred to polydimethylsiloxane (PDMS) and released from AAO template by dissolving aluminum oxide layer in a mixed solution of copper chloride and hydrochloric acid. The branched CNC–PDMS film shows transparent and flexible features with a transmittance of 71% at a wavelength of 550 nm. The organized nanocup layer in the branched CNC film functions as a current collector, while the defective branches on top function act as an electrode. Polyvinyl alcohol-phosphoric acid (PVA-H_3_PO_4_) was used as an ionic polymer electrolyte as well as a separator. In order to obtain an effective electrolyte thickness (12 μm), PVA-H_3_PO_4_ solution was spin-coated on the branched CNC film at 500 rpm.

The rectangular cyclic voltammetry (CV) curve (Figure 5b) was obtained at a very high scan rate (500 mV/s), exhibiting the high performance of branched CNC supercapacitors. Capacitance of 409 μF cm^−2^ was calculated by geometric area from Galvanostatic charge/discharge (CD) curves. When the electrochemically active surface is maximized by modifying CNC film to branched CNC film, the measured specific capacitance is six times higher than the value reported for the single-layer graphene device [54]. The branched CNC supercapacitor device offers higher transparency than the reported value for laser-scribed graphene electrochemical capacitors [55], when using the same electrolyte. By increasing temperature to 80 °C from room temperature, capacitance increased three times (1220 μF cm^−2^) than capacitance at room temperature (Figure 5c,d). The peak volumetric power and energy density of the branched CNC supercapacitor were 19 mW/cm^3^ and 47 μWh/cm^3^, respectively (Figure 5e). The supercapacitor devices exhibited long life cycle stability, even under mechanical stress (45° bending): greater than 84% of the initial capacitance after 10,000 cycles. For the demonstration, a prototype of a large area supercapacitor film (3 cm × 1.5 cm) was manufactured, and the light-emitting diode (LED, working potential 1.5 V) was successfully turned on for 20 min after charging at 2.5 V for 15 min (Figure 5f,g).

The branched CNC film’s unique morphological and structural features allow for excellent conformal packing of polymer electrolytes, maximizing active electrochemical surface area and achieving high energy densities. The device design enables mechanically flexible energy storage devices. It can be integrated into roll-up displays, wearable devices, organic solar platforms, and other unique applications requiring high form factor and light transmission.

## 4. CNTs–CNC Hybrid Structure Electrodes for High Power Supercapacitors

In order to apply CNC films to energy storage devices as an electrode, they need to have high porosity or high surface area to deliver high supercapacitor performance. When electrodes have large surface area like porous carbon electrodes [57,58], the amount of interaction between electrolyte ions and electrodes can be increased. Therefore, carbon nanostructured materials such as activated carbon, carbon nanotubes (multi-walled and single-walled CNTs), carbon nanowire, spherical carbon nanoparticles, and single-layer to few-layered graphene structures are used as main material of electrodes [58,59,60,61,62,63,64]. CNTs–CNC hybrid structure is a combination of a continuous 2D film of carbon nanocups array and a three-dimensional CNT forest on top of CNC. The increased surface area of the CNTs–CNC hybrid structure improves the electrochemical properties of the supercapacitor by providing a large interfacial area. The larger interfacial area provides interactive sites where electrolyte ions can interact with the electrode surface, and CNTs provide high electrical conductivity.

**Figure 6:**
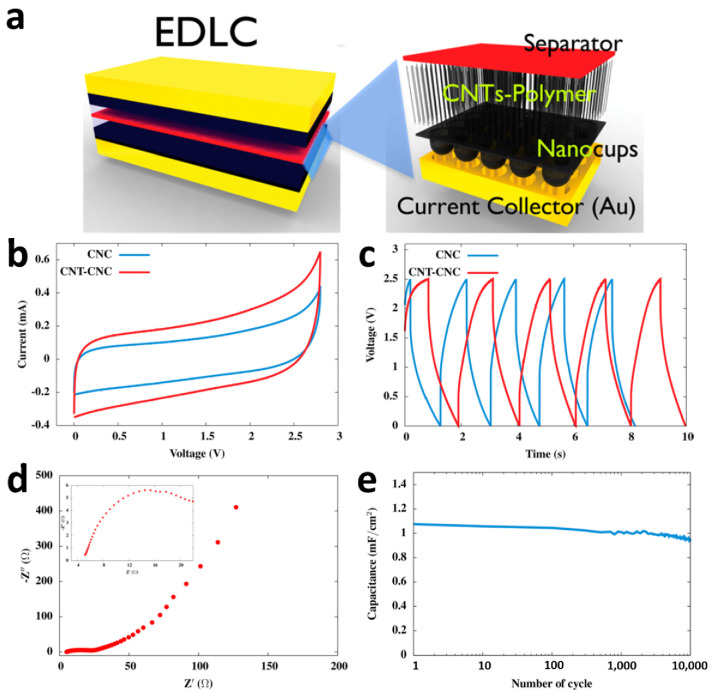
(**a**) Schematic of the supercapacitor consisted of two CNT−CNC hybrid structures on Au current collectors. (**b**) Cyclic voltammograms of supercapacitor cells having CNC and CNT−CNC electrodes, at a scan rate of 1 mV/s in 1 M LiPF6 electrolyte. (**c**) Galvanostatic charge−discharge behavior of supercapacitor cells with CNC and CNT−CNC electrodes, at an applied constant current of 10 μA in 1 M LiPF6 electrolyte. (**d**) Complex-plane impedance spectrum of supercapacitor cell having a CNT−CNC electrode, measured at AC amplitude of 10 mV, in 1 M LiPF6 electrolyte. The inset shows the impedance spectrum of the initial state. (**e**) Areal capacitance vs. cycle number plot of supercapacitors having CNT−CNC electrodes. Reproduced with permission from [36]. Copyright (2012) American Chemical Society.

To fabricate the supercapacitors using CNTs–CNC hybrid structure as an electrode, a thin layer of poly(methyl methacrylate), PMMA, was spin-coated on top of the CNTs–CNC–AAO structure, then AAO template was dissolved by chemical etching. The Au layer was coated on the bottom of the CNC layer after removal of the AAO template, where the layer works as a current collector. The supercapacitor was fabricated by stacking two symmetric electrodes with the filter paper in between (Figure 6a). Both the thin PMMA film and the filter paper work as a polymer electrolyte and separator, respectively. Specifically, 1 M of LiPF_6_ electrolyte in ethylene carbonate and dimethyl carbonate (1:1, v/v) was used to soak both separators, PMMA and filter paper. By soaking in the electrolyte solvent, the polymer separator formed a polymer-gel electrolyte. Since the CNTs inside of the nanocups have a cylindrical shape and can be interpreted as an exohedral type, the CNTs–CNC supercapacitor needs to be considered as a combination of the electric two-cylinder capacitor (EDCC) and electric double-layer capacitors (EDLC).

The electrochemical behavior of CNTs–CNC supercapacitor was tested and compared with the CNC electrode to demonstrate performance enhancement. Figure 6b–e shows the electrochemical properties of CNTs–CNC supercapacitor. The specific capacitances of the CNTs–CNC supercapacitor was calculated as 45 F/g, while that of the CNC supercapacitor was 30 F/g. The capacitance of a CNC supercapacitor is 0.6 mF/cm^2^ when the capacitance is normalized with respect to the geometric area, which is comparable with the report of onion-like carbon electrodes [65]. The areal capacitance was improved to 1.0 mF/cm^2^ by growing of vertically aligned CNTs on CNC. Furthermore, the cyclic stability tests of CNT-CNC supercapacitors show stable capacitance up to 10,000 charge/discharge cycles (Figure 6e). Experimental results show that the three-dimensional CNT-CNC hybrid design nearly doubled the areal specific capacitance of supercapacitors through improved charge accommodation. Figure 6d shows that the nanoscale contact between the CNT-CNC electrode and deposited Au current collector has a low equivalent series resistance of 23 Ω per unit area (m^2^). The CNTs-CNC supercapacitors showed improved specific and areal capacitance when compared to the CNC supercapacitors. By increasing the specific surface area via the growth of CNTs on the CNC layer, the capacitance and the supercapacitors’ cell performance significantly improved.

## 5. CNC Container System for Liquid Metal Nanodroplet Dynamics Study

CNC can be engineered to be an effective nanocontainer system due to its open nanoscale cup morphology, strong C-C covalent bonds, and multilayered graphitic nature. The low aspect ratio open cup morphology and the graphitic nature makes CNC structure a powerful template for container systems at nanoscale. With strong C-C covalent bonds, CNC is robust to prevent change in volume and interfacial area upon heating. Various metals have been successfully inserted inside the nanocups using the evaporation method, followed by thermal annealing process [27].

The dynamic movements of molten Pb nanoparticles in CNC containers were directly observed using in-situ TEM. It shows random collective motions driven by collisions when the lead (Pb) metal is trapped inside the sealed CNC nanocontainers and is thermally activated. CNC containers were able to withhold increases in metal particles’ size and their drastic changes in mobility. The size of Pb nanoparticles inside the CNC containers can be precisely controlled by the deposition time of Pb. Then, the free space inside of CNC containers can be calculated, and the motion of nanodroplet can be determined when heat is applied.

**Figure 7:**
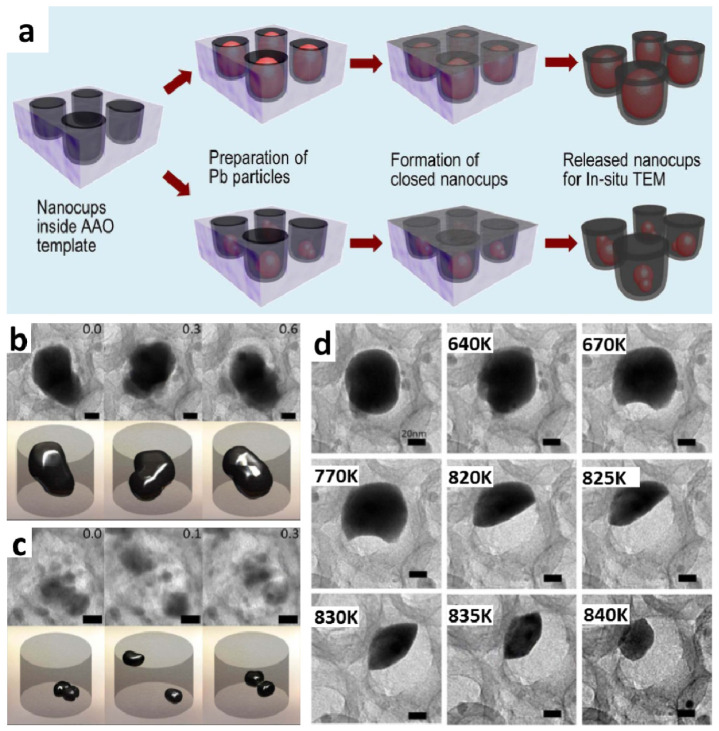
(**a**) Schematic of the overall process in making a closed CNC container system filled with different-sized Pb nanoparticles. (**b**) Large size Pb nanodroplet at 630 K inside nanocontainer. (**c**) Two small Pb nanodroplets at 600 K inside nanocontainers. (**d**) The size of Pb nanodroplet gradually decreases in the open CNC container due to evaporation. Originally, at 640 K, the liquid Pb nanodroplet stopped motion and became spherical. After 0.03 s, this stationary Pb droplet moved towards the edge of the nanocontainer. At 670 and 770 K, the nanodroplet formed a meniscus shape. Eventually, the liquid Pb changed to a hollow, concave shape at 820, 825, 830, 835, and 840 K, respectively. All scale bars are 20 nm. Reproduced with permission from [38]. Copyright (2013) Springer Nature.

The fabrication process of Pb nanoparticles in the CNC system is illustrated schematically in Figure 7a. CNC nanocontainer was fabricated using precisely controlled low aspect ratio nanopores inside the AAO templates, followed by a thermal chemical vapor deposition of acetylene (C_2_H_2_) at 650 °C and Ar ion milling processes [27,37]. Different-sized Pb nanoparticles were inserted in the nanocup container by controlling the thickness of Pb deposition and thermal annealing in the Argon environment. During the annealing process, thermal re-evaporation occurred at the deposited metal layer inside the nanocups, and Pb nanoparticles were formed inside the nanocontainer. Finally, the nanocup containers with Pb nanoparticles were sealed with an amorphous carbon layer at the open end, constructing a closed nanocup system.

In situ TEM studies were performed to directly observe the motions of Pb nanodroplets in the sealed CNC containers. The temperature of the CNC container to complete solid-to-liquid phase transition increased to 630 and 600 K for large Pb nanoparticles (60 nm in diameter) and small Pb nanoparticles (15–20 nm in diameter), respectively, as confirmed by molecular dynamics simulations. As shown in Figure 7b, at the early period of heating, larger Pb nanoparticles melted and started to fluctuate in both radial and axial directions inside the cylindrical CNC container. This motion became more significant along the axial direction over time. Meanwhile, smaller Pb nanodroplets showed constant movement inside the nanocontainer as shown in Figure 7c. Compared with the motion of the larger droplets, individual movements of smaller nanodroplets appeared more random at much faster rate. The linear increase in the mean square displacement (MSD) of their position in two-dimensional space is indicative of the Brownian dynamics for the smaller droplets. The droplets remain active only in closed environments, and they become immobile and likely to evaporate to the outside vacuum environment when in open CNC containers (Figure 7d).

The difference in the mobilities and the diffusion properties of metal nanoparticles appear to be a function of their size, wetting, and constraints. The particles remain active only in a closed environment, and once exposed to vacuum, they become vulnerable to evaporate. The results indicate that in a closed state, nanodroplets undergo Brownian motion, maintaining high mobility. The study of metal nanodroplets constrained in both closed and open CNC containers are important for understanding the effects of constraining the activity of nanostructures in environments such as porous structures. The CNC system shows high potential to be used as a nanoscale container for both open and closed structure in various applications such as drug delivery, sensors containing nanoparticles, and nanoscale multicomponent systems.

## 6. Conclusions and Future Perspectives

Low aspect ratio graphitic nanoscale architecture based on CNC film exhibits high potential in various applications due to excellent features such as high transparency, flexibility, and large specific surface area. The morphology of the film can be easily modified by forming branches at the bottom of the CNC or by growing CNT forests on the top of the CNC film. This review demonstrated the successful use of highly engineered CNC film as electrodes for flexible, transparent, and high-performance supercapacitors. Both the branched CNC and the CNTs–CNC hybrid structure showed higher performance and capacitance over the standard CNC film. The standard CNC film can be further tailored to achieve adaptability for various applications. This paper also showed the study of the dynamics of liquid metal nanodroplets in the CNC container system. The CNC film may be used as a container for multicomponent structures, e.g., as applications in surface-enhanced Raman scattering (SERS), nanomedicine, and nanometrology.
